# Supplementary material for: The Synergistic Effect of Co-Treatment of Methyl Jasmonate and Cyclodextrins on Pterocarpan Production in Sophora flavescens Cell Cultures
Source: Int J Mol Sci. 2020 May 30;21(11):3944. doi: 10.3390/ijms21113944 (PMC7313034; doi:10.3390/ijms21113944)
Supplement: Supplementary file 1 [file ijms-21-03944-s001.pdf]

Supporting Information

Supplementary Materials

Table S1. Primer pairs used for qRT-PCR in this study.

Gene	Gene name	Gene information	Primer sequences (forward/reverse)
SfPAL	Phenylalanine ammonia lyase	Sf102447_c0_g1_i1	5′-ATCTCTCAGGTGGCTGCCAT-3′<br>5′-TGCCTGAGTACCCTTGGAGG-3′
SfC4H	Cinnamate-4-hydroxylase	Sf110063_c0_g1_i1	5′-ACCATGCACTTAGCCTGATG-3′<br>5′-AGAGATGGGGAAAGTGGCA-3′
Sf4CL	4-Coumarate-CoA-ligase	Sf125371_c0_g2_i1	5′-AACAGGGTTGCCAAAAGGGG-3′<br>5′-GGGGAATTTGGCCCTGACAG-3′
SfCHS	Chalcone synthase 3	MH402001.1	5′-GGAAAGAAGCTGCAACCAAG-3′<br>5′-TCTTCACATAAGGGCGAAGG-3′
SfCHR	Chalcone reductase 3	MH450276.1	5′-TCCTCCTTCGGTCAATCAAG-3′<br>5′-TCCCAAAGGAGAGAATGCAG-3′
SfCHI	Chalcone isomerase	Sf127219_c1_g1_i1	5′-TCTGCAAATTGTTCAATGGC-3′<br>5′-CCCCTCCAGGATCCTCCAAG-3′
SfIFS	Isoflavone synthase 1	MH538346.1	5′-TCGTCCTGAAAGGTTCTTGG-3′<br>5′-AATTCACTCCAGGGCACATC-3′
SfI3'H	Isoflavone 3'-hydroxylase	Sf64464_c0_g1_i1	5′-CTGTGCCAGCTTCTGCATCA-3′<br>5′-ATTCCCTCTGGTTCGGCTCA-3′
SfIFR	Isoflavone reductase	MH450252.1	5′-AGCCATTGGAAGGCACATAG-3′<br>5′-AGCAGCTCTTGCTTGGTTTC-3′
SfIF7GT	UDP-glucose: isoflavone 7-*O*-glucosyl transferase	DN13644_c1_g2.p1	5′-CACTCACGAACCCATCTTTG-3′<br>5′-CGAAGGAGAAAGGAATGGTG-3′
SfACT11	Actin11	Sf128341_c1_g1_i1	5′-TTGGTCGACCTCGCCATACT-3′<br>5′-GGGAGAGGACAGCCTGGATT-3′

Primers were designed using the Beacon designer™ software (Premier Biosoft)

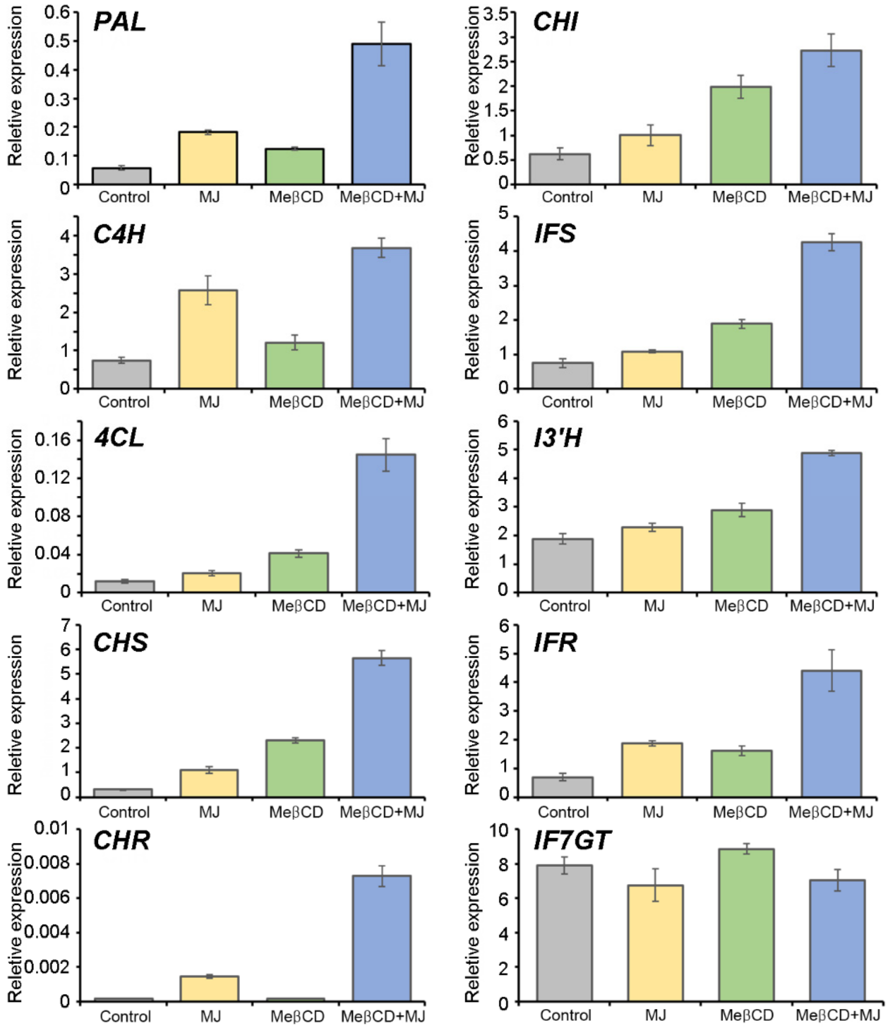

Figure S1. Relative expression of pterocarpan biosynthetic genes in the *S. flavescens* cells elicited by MJ, Me CD, and MJ+Me CD. Transcript levels of *PAL, C4H, 4CL, CHS, CHR, CHI, IFS, I3'H, IFR*, and *IF7GT* were analyzed by qRT-PCR using the *S. flavescens* cells elicited with 50 µM MJ, 50 mM Me CD, and 50 µM MJ+50 mM Me CD for 24 h. The relative expression levels were normalized to the level of actin (*SfACT11*, transcript ID: *Sf128341_c1_g1_i1*) as a quantitative control and were presented as the fold induction relative to actin. Data are the mean of three independent replicates ± SD.

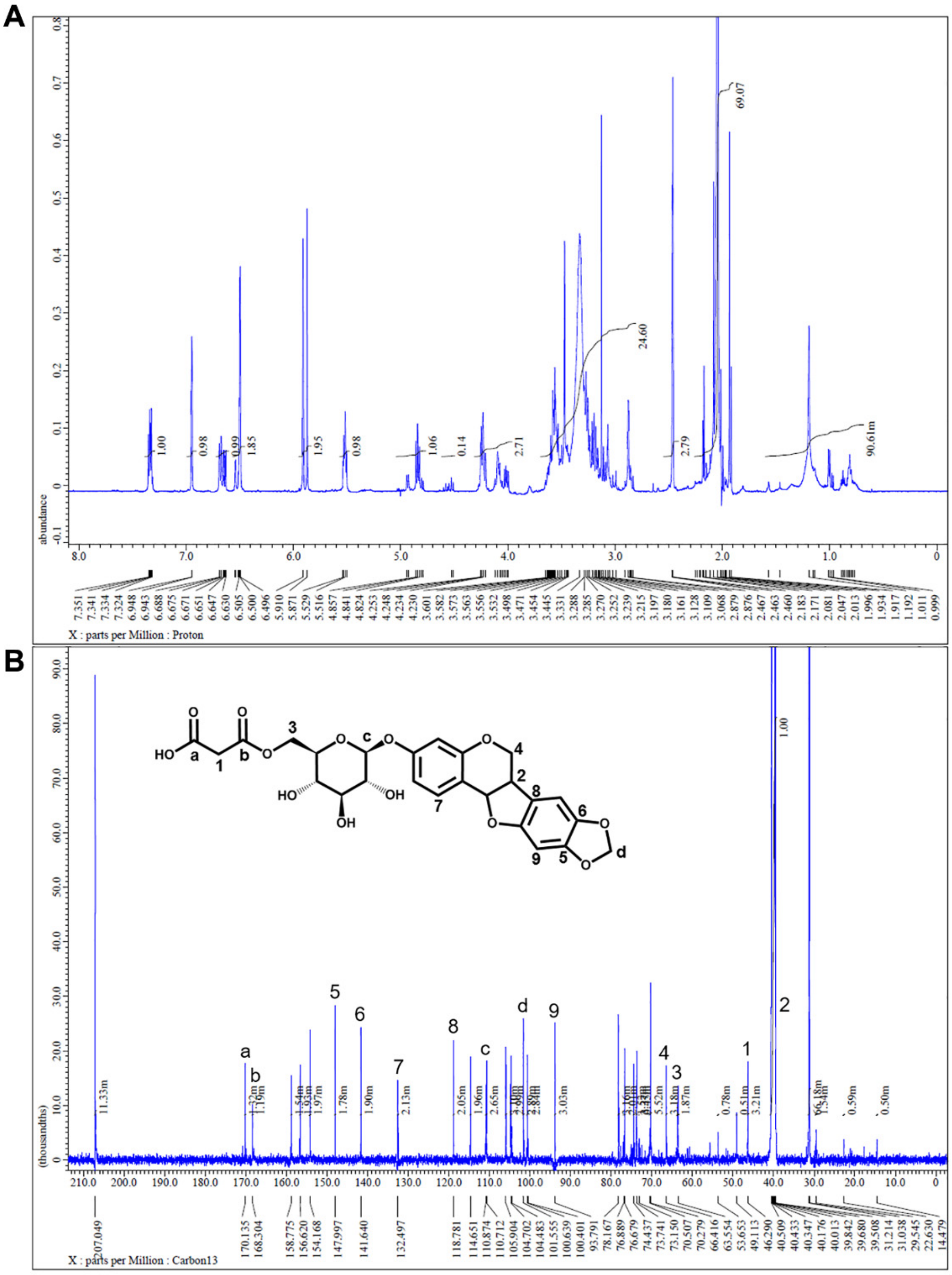

Figure S2. ^{1}H- and ^{13}C-NMR spectral data (DMSO solution) of trifolirhizin malonate identified from the *S. flavescens* cell cultures. Trifolirhizin malonate: (**A**) ^{1}H-NMR (500 MHz, DMSO-d_6) δ 7.34 (1H, *d*, *J* = 9.0 Hz), 6.94 (1H, s), 6.69 (1H, *dd*, *J* = 2.5, 9.0 Hz), 6.50 (2H, *d*, *J* = 2.5, 5.0 Hz), 5.91 (2H, *d*, *J* = 18.5 Hz), 5.51 (1H, *d*, *J* = 4.0 Hz), 4.83 (1H, *d*, *J* = 7.5 Hz), 4.24 (1H, *d*, *J* = 6.0 Hz), 4.09 (2H, m), 3.57~ 3.06 (6H, m), 2.88 (2H, s); (**B**) ^{13}C-NMR (125 MHz, DMSO-d_6) δ 170.135 (C-a), 168.3 (C-b), 147.9 (C-5), 141.6 (C-6), 132.4 (C-7), 118.7 (C-8), 110.7 (C-c), 101.5 (C-d), 93.7 (C-9), 66.4 (C-4), 63.5 (C-3), 46.2 (C-1), 40.1 (C-2).
